# Large-scale bulk RNA-seq analysis defines immune evasion mechanism related to mast cell in gliomas

**DOI:** 10.3389/fimmu.2022.914001

**Published:** 2022-09-08

**Authors:** Rui Chen, Wantao Wu, Tao Liu, Yihan Zhao, Yifan Wang, Hao Zhang, Zeyu Wang, Ziyu Dai, Xiaoxi Zhou, Peng Luo, Jian Zhang, Zaoqu Liu, Li-Yang Zhang, Quan Cheng

**Affiliations:** ^1^ Department of Neurosurgery, Affiliated Nanhua Hospital, University of South China, Hengyang, China; ^2^ Department of Oncology, Xiangya Hospital, Central South University, Changsha, Hunan, China; ^3^ National Clinical Research Center for Geriatric Disorders, Xiangya Hospital, Central South University, Changsha, China; ^4^ Department of Neurosurgery, Xiangya Hospital, Central South University, Changsha, China; ^5^ Xiangya School of Medicine, Central South University, Changsha, China; ^6^ Department of Oncology, Zhujiang Hospital, Southern Medical University, Guangzhou, China; ^7^ Department of Interventional Radiology, The First Affiliated Hospital of Zhengzhou, Zhengzhou, China

**Keywords:** mast cell, immunotherapy, glioma microenvironment, immune evasion, prognostic model

## Abstract

Accumulating evidence has demonstrated that the immune cells have an emerging role in controlling anti-tumor immune responses and tumor progression. The comprehensive role of mast cell in glioma has not been illustrated yet. In this study, 1,991 diffuse glioma samples were collected from The Cancer Genome Atlas (TCGA) and the Chinese Glioma Genome Atlas (CGGA). xCell algorithm was employed to define the mast cell-related genes. Based on mast cell-related genes, gliomas were divided into two clusters with distinct clinical and immunological characteristics. The survival probability of cluster 1 was significantly lower than that of cluster 2 in the TCGA dataset, three CGGA datasets, and the Xiangya cohort. Meanwhile, the hypoxic and metabolic pathways were active in cluster 1, which were beneficial to the proliferation of tumor cells. A potent prognostic model based on mast cell was constructed. *Via* machine learning, DRG2 was screened out as a characteristic gene, which was demonstrated to predict treatment response and predict survival outcome in the Xiangya cohort. In conclusion, mast cells could be used as a potential effective prognostic factor for gliomas.

## Introduction

Gliomas are one of the most common primary malignant tumors, accounting for 80% of all brain malignant tumors (1, 2). Gliomas are usually characterized by abnormal invasion and destruction of the blood–brain barrier (3, 4). At present, the main clinical treatments for gliomas are surgical resection, chemotherapy, and radiation, but their therapeutic effect remains unsatisfactory (5). Since the theory that the brain has absolute immune privilege has been questioned and denied, immunotherapy for brain tumors has been vigorously developed (6, 7). At present, the research direction of immunotherapy for gliomas mainly includes active immunotherapy and systemic or local delivery of immunomodulators (8). A deeper understanding of the tumor microenvironment (TME) of gliomas may help the development of immunotherapy.

In recent years, immune cells in TME have been considered as important targets of tumor immunotherapy (9). Mast cells are one of the early infiltrating cells before tumorigenesis, and play a crucial role in tumor angiogenesis and remodeling TME in gliomas (10, 11). It has been reported that mast cells within the tumor differ significantly in protease profiles or subtypes from mast cells outside the tumor (12). In TME, mast cells will become highly proinflammatory and actively recruit macrophages and other innate immune cells after activation and degranulation to coordinate the anti-tumor immune response (13). Similar to macrophages, the role of mast cells in tumors remains controversial because mast cell-related inflammatory processes can both promote or inhibit tumor development (14). Some studies have proposed that mast cells could be transformed into different phenotypes to exert different effects, and this transformation can be co-regulated by macrophages and tumor cells (15). In gastric cancer, a linear signaling axis activated by tumor epithelial-derived IL-33 was found to activate mast cells and promote tumor-associated macrophage (TAM) accumulation. The accumulation of TAMs was associated with inferior survival in patients with gastric cancer (16). In addition, the role of mast cell-derived histamine and ATP in secretory and phagocytic regulation may explain the heterogeneity of microglial responses (17). In the studies of colon carcinoma, mast cells have also been found to enhance the immunosuppressive properties of MDSCs through the production of IFN, and the M2-type tumor-associated macrophage is a major source of MDSCs (18). Hence, the role of mast cells in TME may be related to TAMs.

In this study, we used xCell algorithm to identify meaningful mast cell-related genes in gliomas and to divide glioma samples into two clusters with different tumorigenic and immunogenic characteristics. A risk score predicting malignancy of gliomas and poor prognosis of glioma patients was further constructed to predict the efficacy of immunotherapy.

## Method

### Patient and cohort inclusion

We collected diffuse glioma samples from two datasets based on The Cancer Genome Atlas (TCGA) and the Chinese Glioma Genome Atlas (CGGA). The TCGA cohort includes glioma samples. The RNA-seq data and corresponding clinical information are retrieved from the TCGA dataset (http://cancergenome.nih.gov/). In this study, we used two RNA-seq cohorts (CGGA325 and CGGA693) and a microarray cohort (CGGAarray) as validation sets. The RNA-seq and microarray data, and clinical and survival information are retrieved from the CGGA dataset (http://www.cgga.org.cn).

### Identification of mast cell-related genes

The xCell algorithm defines mast cells in the TCGA dataset (19). In TCGA and three CGGA cohorts, mast cell-related genes with a correlation efficiency > 0.4 were screened out, and the gene matrix was crossed to obtain 495 mast cell-related genes. After performing univariate Cox regression analysis, 280 genes were proved to be prognostic genes.

### Construction of mast cell-related subtypes

Based on 280 prognostic genes related to mast cells, we identified the robust clusters of glioma patients from the TCGA by using the consensus clustering algorithm of partition around medoids (PAM). After intersecting the 280 prognostic genes with the gene expression profiles from CGGA325, CGGA693, and CGGAarray datasets, 248 prognostic genes were used for identifying the robust clusters of glioma patients in three CGGA datasets using PAM. Then, we used the cumulative distribution function (CDF) and consensus heatmap to evaluate the optimal K value of 2.

### Annotation of the immune infiltrating microenvironment

ESTIMATE is used to score the immune cell infiltration level (immune score) and stromal content (stromal scores) of each sample. We used the xCell algorithm (19) to quantitatively analyze the enrichment levels of 64 immune signals, and used the CIBERSORT algorithm (20) to estimate the relative scores of 22 immune cell types in tumor tissues. The GO pathway was studied by performing gene set variation analysis (GSVA), and GO items with a *p*-value <0.05 were screened out. From previous studies, seven classifications of immunomodulators were analyzed (21, 22).

### Identification of an immune-related signature

Then, we use elastic regression analysis and PCA based on the 248 prognostic genes to further calculate the patient’s risk score. Twenty-nine genes were included for the construction of the risk score. The extracted principal component 1 is used as the signature score. The risk score after the prognostic value of the genetic signature score of each patient is obtained by the following formula:


$$\text{risk score}=\sum PC1_{i},$$

where *i* represented the expression of genes.

### Prediction of immunotherapy responses

The IMvigor210 cohort is a cohort of urothelial cancer treated with the anti-PD-L1 antibody atezolizumab, which can be used to predict the therapeutic effect of immunotherapy on patients (22, 23). Based on the Creative Commons 3.0 license, all clinical data and expression data were downloaded from http://research-pub.Gene.com/ IMvigor210CoreBiologies. The DEseq2 R software package (24) was used to standardize the raw data.

### Construction and validation of a prognostic model

We use nomograms to visualize multi-factor regression analysis, which is usually used for cancer survival rate prediction. The risk score groups, age, pathological stage, and mutation status of glioma were selected to construct the variables of the nomogram, and univariate and multivariate regression analyses were used to evaluate the prognostic value of these factors.

### RNA sequencing of the Xiangya cohort

Tumor tissues from 105 glioma patients who underwent surgical resection in Department of Neurosurgery, Xiangya Hospital were collected for sequencing.Glioma tissues were collected and written informed consent was obtained from all patients. The included glioma tissues were approved by the Ethics Committee of Xiangya Hospital, Central South University. The detailed procedure was reported in our previous findings (25–27). The survival information of the patients was collected for conducting the survival analysis. The mast cell density in the Xiangya cohort was calculated using the xCell algorithm. The risk score was independently calculated in the Xiangya cohort.

### Statistical analysis

The Kaplan–Meier curves with log-rank test were used to evaluate the survival difference between the two groups, and all survival curves were generated using the R package survminer. Prognostic factors were assessed by univariate and multivariate Cox regression analysis. The OS and risk scores were calculated based on the R package survival, and we used the R package ggplot2 to visualize the data. The heatmap is generated using pheatmap. For normally distributed variables, significant quantitative differences between and among groups were determined by a two‐tailed t-test or one‐way ANOVA, respectively. For nonnormally distributed variables, significant quantitative differences between and among groups were determined by a Wilcoxon test or a Kruskal–Wallis test, respectively. All statistical analysis was performed using R software. *p* < 0.05 is statistically significant.

## Results

### TME characteristics of the mast cell-stratified groups

**Figure 1:**
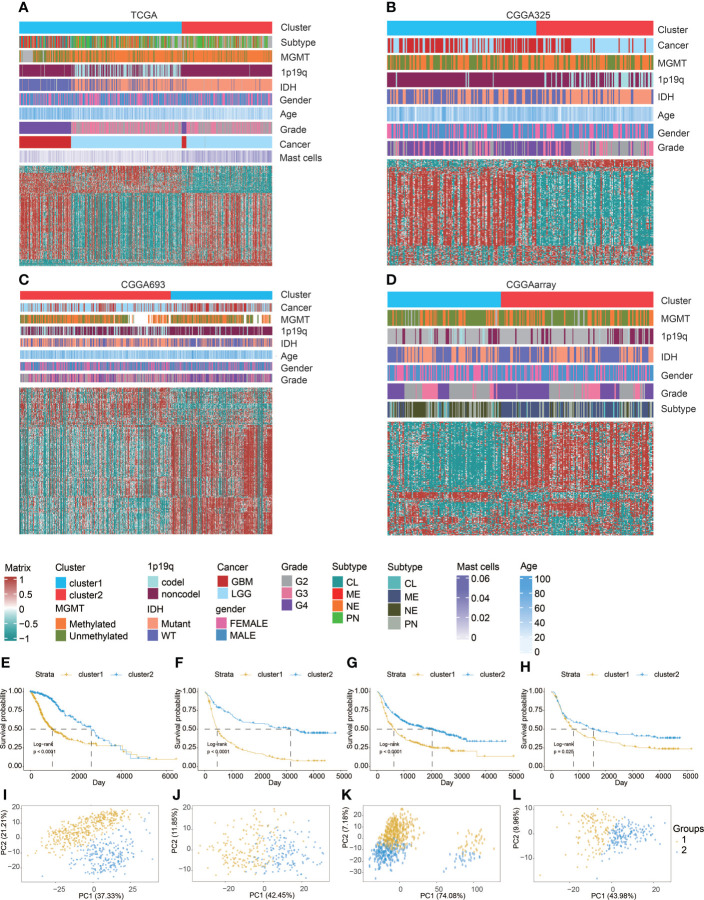
Machine learning for validation of clustering based on mast cell-related genes. Clustering heatmaps demonstrating good separation of the two clusters by traits in **(A)** TCGA, **(B)** CGGA325, **(C)** CGGA693, and **(D)** CGGAarray. Kaplan–Meier survival analysis of the two clusters in **(E)** TCGA, **(F)** CGGA325, **(G)** CGGA693, and **(H)** CGGAarray. Sample clustering by PCA in **(I)** TCGA, **(J)** CGGA325, **(K)** CGGA693, and **(L)** CGGAarray.

**Figure 2:**
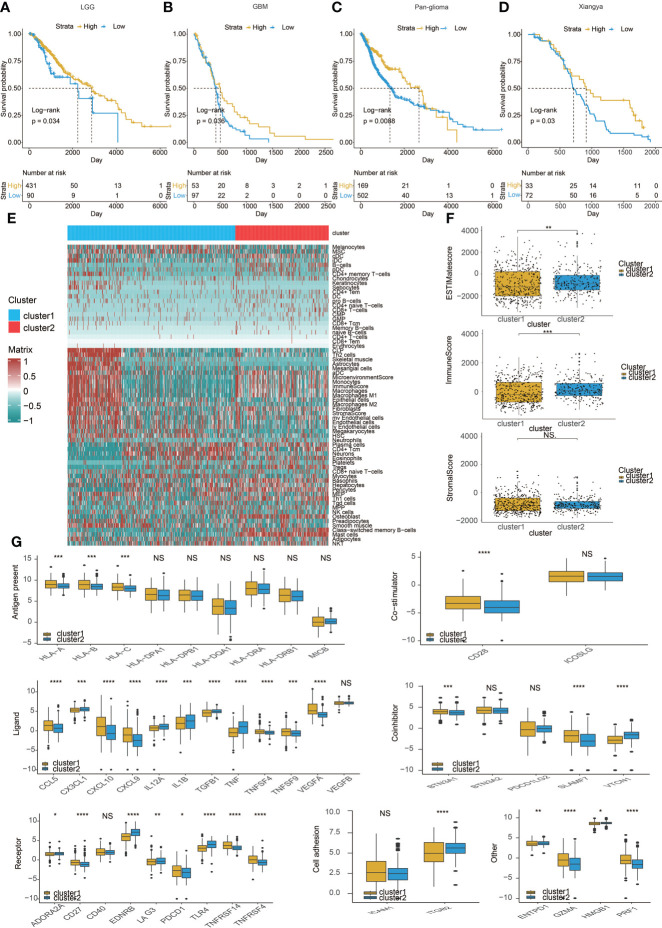
Immune characteristics of the two clusters. Kaplan–Meier analysis of overall survival (OS) based on high vs. low level of mast cell in **(A)** LGG,**(B)** GBM, and **(C)** pan-glioma patients in TCGA. **(D)** Xiangya cohort. **(E)** Heatmap correlating the levels of 64 cell types and clusters in TCGA.**(F)** ESTIMATEScores, ImmuneScores, and StromalScores of the two clusters in TCGA. **(G)** Molecule levels of immune checkpoints in two clustersin TCGA. *p < 0.05, **p < 0.01,***p < 0.001, ****P < 0.0001. NS, not statistically significant.

We used partition around medoids (PAM) to analyze the gene expression profiles of glioma patients in the TCGA dataset (**Figure 1A**) and three CGGA datasets (**Figures 1B-D**), which showed different levels of mast cells and clinical characteristics between the groups. Subsequently, we used the ConsensusClusterPlus package (28) to calculate the optimal number of clusters, and the results showed that the stability of the clustering results was optimal when the number was equal to 2 (Figure S1). The survival analysis of cluster 1 and cluster 2, respectively, confirmed that the prognosis of cluster 1 was worse (**Figures 1E-H**). PCA tried to differentiate the samples from the TCGA dataset (**Figure 1I**) and three other CGGA datasets (**Figures 1J-L**). In addition, we divided patients with different levels of mast cell into high and low levels. Survival analysis also showed that patients with low mast cell level in LGG, GBM, pan-glioma, and Xiangya cohorts had lower probability of survival (**Figures 2A-D**).

Therefore, we studied the characteristics of the immune microenvironment of the two clusters and analyzed the differences in immune cell components of different clusters in the TCGA (**Figure 2E**) and three CGGA datasets (**Figures S2A–C**). At the same time, we also used CIBERSORT (20) to further compare the differences in immune cells between the two clusters (**Figures S2D, S3A, S4A,** and **S5A**). Moreover, we calculated ESTIMATEScores, ImmuneScores, and StromalScores between the two clusters, but the results were not consistent (**Figures 2F**, **S3B, S4B,** and **S5B**). Finally, we compared a series of immune checkpoint molecular differences related to antigen presentation, co-stimulation, ligand, and so on. We found that most immune checkpoint molecules tend to overexpress in cluster 1 (**Figures 2G**, **S3C, S4C,** and **S5C**).

### Clinical traits of the mast cell-stratified groups

**Figure 3:**
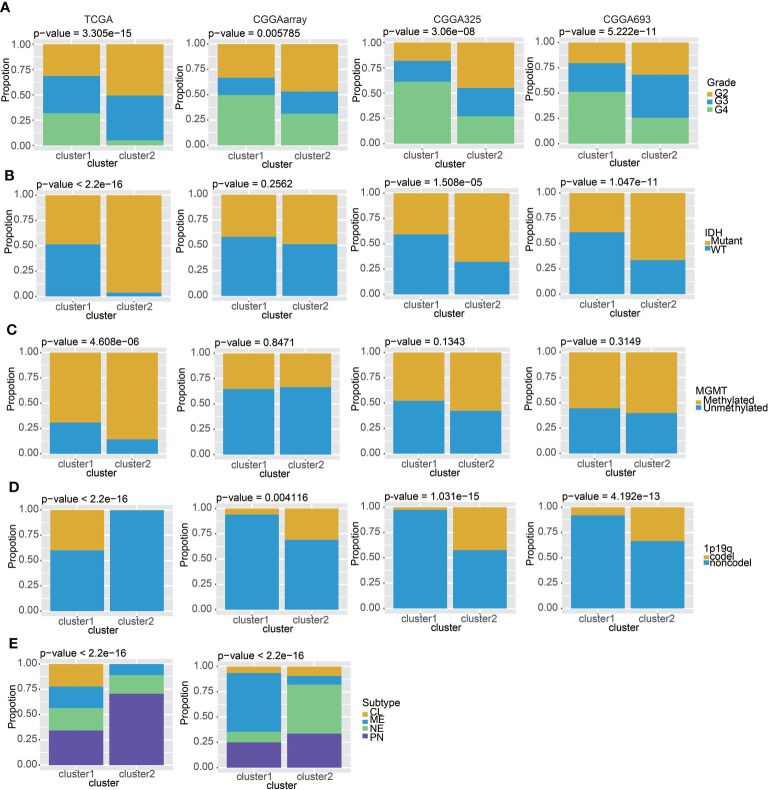
Clinical features of the two clusters. **(A)** The proportions of different tumor grades in TCGA, CGGAarray, CGGA325, and CGGA693. **(B)** Samples with or without the IDH mutation in TCGA, CGGAarray, CGGA325, and CGGA693. **(C)** Samples with or without the MGMT promoter methylation in TCGA, CGGAarray, CGGA325, and CGGA693. **(D)** Samples with or without the chromosome 1p/19q codeletion in TCGA, CGGAarray, CGGA325, and CGGA693. **(E)** The four GBM subtypes in the two clusters in TCGA and CGGAarray.

We studied the differences between cluster 1 and cluster 2 in pathological grade, IDH, MGMT, 1p19q, and glioma subtypes. The results in TCGA and the three CGGA datasets all suggested that gliomas in cluster 1 had a higher pathological grade (**Figure 3A**), and cluster 2 had lower levels of IDH WT (**Figure 3B**) and MGMT promoter unmethylation (**Figure 3C**). It is worth noting that the proportion of samples with chromosome 1p19q codeletion in cluster 1 is higher than that in cluster 2 in the TCGA dataset, but the codeletion ratio in cluster 2 is higher in three CGGA datasets (**Figure 3D**). This may be related to the geographical differences of patients. In addition, we found that, in cluster 1 samples, CL (Classic) and ME (Mesenchymal) subtypes accounted for the majority, while in cluster 2, NE (neural) and PN (pro-neural) subtypes were more common (**Figure 3E**). All above conclusions show that gliomas in cluster 1 are more malignant, which may reflect a worse prognosis.

**Figure 4:**
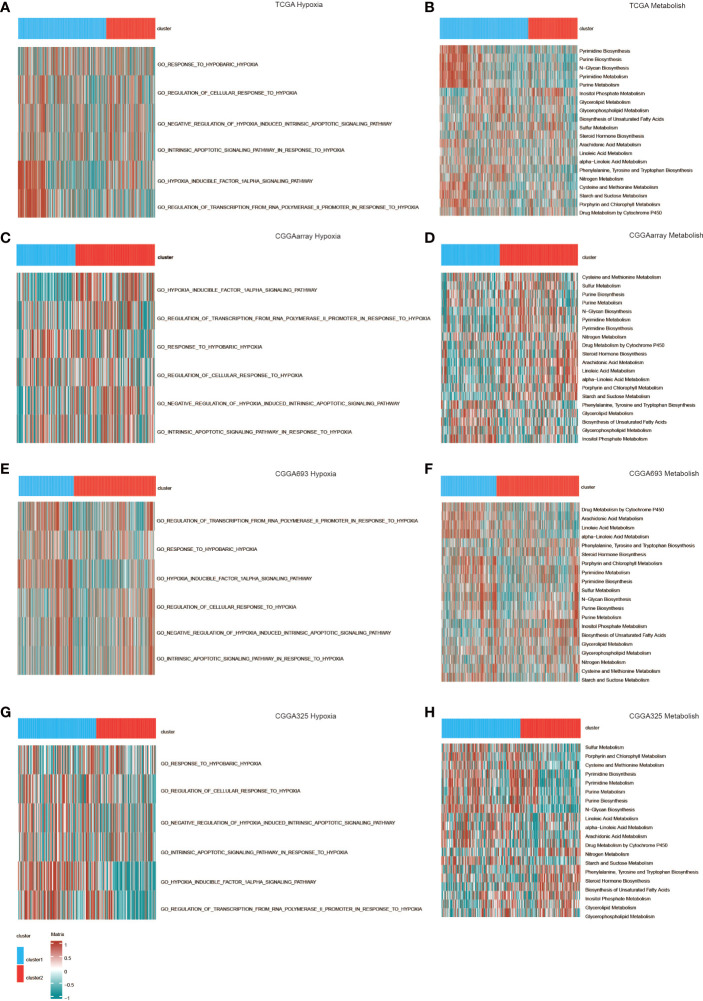
GO functional enrichment analysis of hypoxia and metabolism in the two clusters in **(A, B)** TCGA, **(C, D)** CGGAarray, **(E, F)** CGGA693, and **(G, H)** CGGA325.

We also used GSVA to study the differences in the activation of hypoxia and metabolic pathways between the two clusters. Various hypoxia-related pathways such as the response to hypoxia and the regulation of the cellular response to hypoxia were activated in cluster 1, reflecting the hypoxic state of gliomas. Similarly, cluster 1 also showed excessive activation of metabolic pathways (**Figure 4**). These are signs of the proliferation of malignant tumors, showing the stronger proliferation activity and malignant tendency of glioma in cluster 1.

### Genomic features of the two clusters

**Figure 5:**
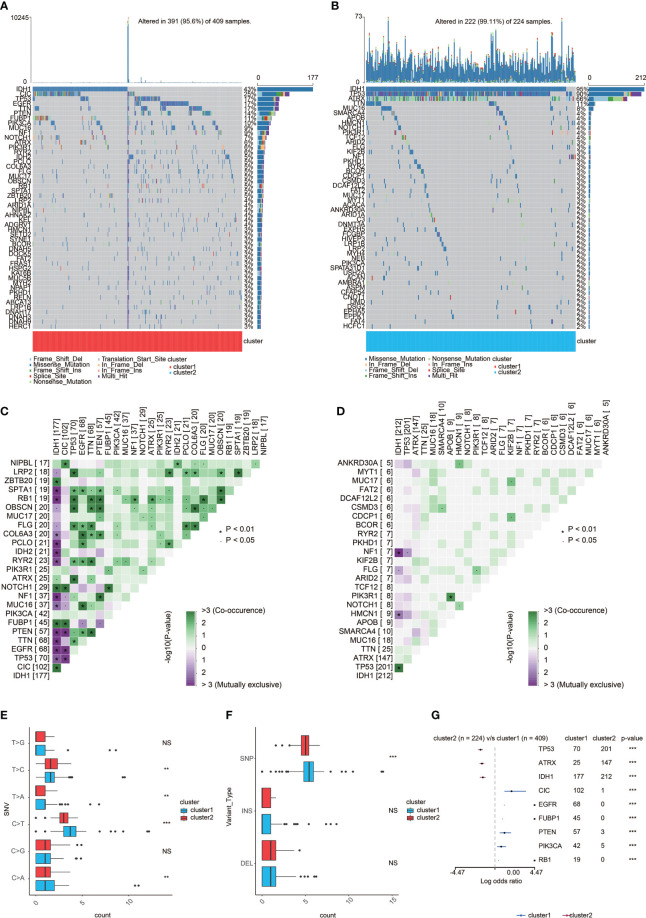
Genomic features of the two clusters. **(A)** List of the most frequently altered genes in cluster 1. **(B)** List of the most frequently altered genes incluster 2. **(C)** The heatmap showing the concurrence or mutual exclusivity of the top 25 most mutated genes in cluster 1. *p < 0.05, *p < 0.01,***p < 0.001, ****p < 0.0001. NS, not statistically significant. **(D)** The heatmap showing the concurrence or mutual exclusivity of the top 25 mostmutated genes in cluster 2. *p < 0.05, *p < 0.01, ***p < 0.001. NS, not statistically significant. **(E)** Frequency comparison of SNVbetween the two clusters. **(F)** Frequency comparison of variant type between the two clusters. **(G)** The Forest plot listing the top nine mostmutated genes between the two clusters. *p < 0.05, **p < 0.01,***p < 0.001. NS, not statistically significant.

According to somatic mutation analysis, mutations in IDH1 (43%), CIC (25%), TP53 (17%), and EGFR (17%) were most highly enriched in cluster 1 (**Figure 5A**). In comparison, IDH1 (95%), TP53 (90%), and ATRX (66%) mutations were enriched in cluster 2 (**Figure 5B**). Missense mutation was the uppermost gene alteration type in all these genes except for ATRX, the strongest co-occurrent pairs of gene alteration included ATRX-TP53 and CIC-IDH1 in cluster 1, and TP53-IDH1 in cluster 2. In addition, the most mutually exclusive pairs were PTEN-IDH1, EGFR-IDH1, PTEN-CIC, and EGFR-CIC in cluster 1, and NF1-IDH1 in cluster 2 (**Figures 5C, D**). Among the detected SNVs, C>T appeared to be the most common mutation in cluster 1 and have a significant higher frequency in cluster 1 (**Figure 5E**). While the frequencies of insertion and deletion were not statistically different between the two clusters, SNP was significantly more common in cluster 1 (**Figure 5F**). The top nine most differentially mutated cancer-related genes are listed in **Figure 5G**.

### Generation of risk score and its functional annotation

**Figure 6:**
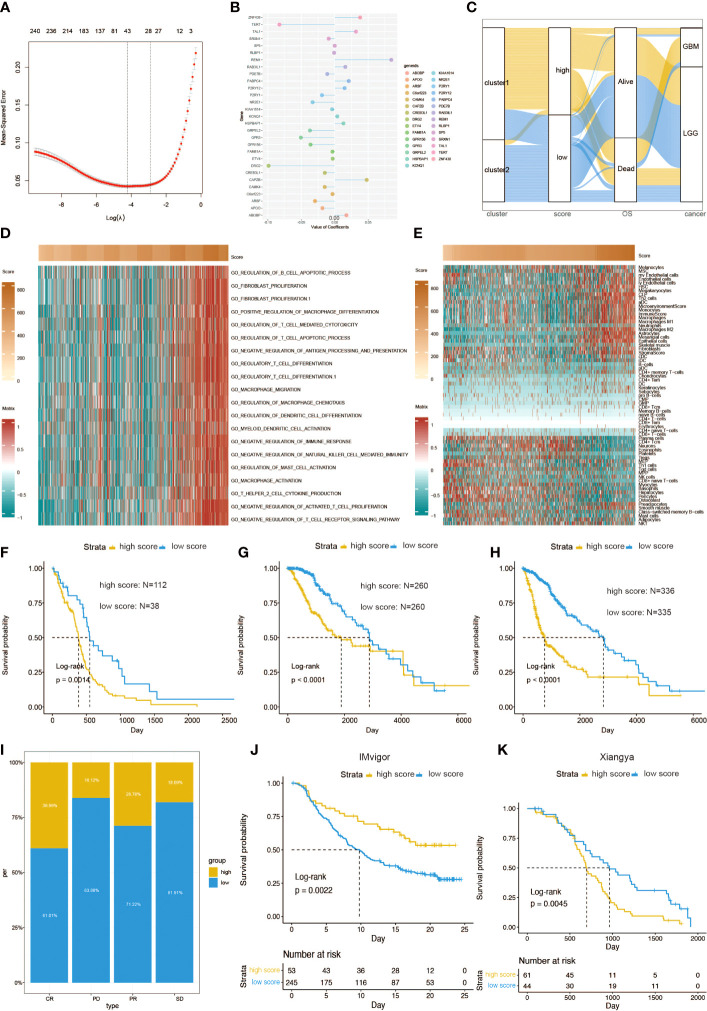
Functional annotation of risk scores. **(A)** Elastic regression analysis was performed to screen out the prognostic genes. **(B)** Elastic net regression analysis and PCA obtained 30 mast cell-related genes and their coefficients. **(C)** A Sankey plot was used to reveal the correlation between cluster, scores, OS, and cancer types. **(D)** GO functional enrichment analysis correlating different immune regulatory processes with risk score. **(E)** Heatmap correlating the risk score and 64 cell types. Survival curves of risk scores in **(F)** GBM, **(G)** LGG, and **(H)** pan-glioma patients. **(I)** The percent of different risk score in CR, PD, PR, and SD of glioma patients. **(J)** Kaplan–Meier analysis of survival probability based on high *vs*. low risk score from the IMvigor210 cohort. **(K)** Kaplan–Meier analysis of survival probability based on high *vs*. low risk score from the Xiangya cohort.

By conducting elastic net regression analysis (**Figure 6A**), we obtained the 29 most important genes and their coefficients from 248 prognostic genes for the construction of a mast cell-related risk signature (**Figure 6B**). Sankey plot revealed a high degree of consistency between mast cell-related clusters and risk scores (**Figure 6C**). Pathways related to macrophage migration and activation, regulation of mast cell activation, fibroblast proliferation, and the Th2 cell cytokine production were more active in the samples with higher scores (**Figure 6D**). The correlation between the expression level of 64 kinds of cells and risk scores was evaluated. The risk score was positively correlated with the levels of fibroblasts, macrophages, and Th2 cells, and negatively correlated with mast cells and Th1 cells (**Figure 6E**). In addition, the risk score is also related to immune checkpoint molecules. Similar to cluster 1, gliomas with high scores tend to express higher levels of immune checkpoint molecules (Figure S6). In the TGCA dataset, survival analysis showed that patients with different mortality risks in LGG, GBM, and pan-glioma were well separated by high and low risk scores (**Figures 6F–H**). According to the risk scores for the immunotherapeutic response types of patients with urothelial carcinoma, CR and PR seemed to be more likely to have lower risk scores (**Figure 6I**). We evaluated the efficacy of using risk scores to predict the prognosis of immunotherapy. Patients can be stratified according to high and low risk scores in the IMvigor210 (**Figures 6J**). Patients can also be stratified according to high and low risk scores in the Xiangya cohort (**Figures 6K**).

### Construction of a prognostic nomogram based on risk scores

A prognostic nomogram was constructed to further investigate the predictive efficiency of mast cell density. The construction of this nomogram has taken into account several prognostic factors, such as risk score groups, patient age, glioma grades, IDH mutation, and chromosome 1p/19q codeletion (**Figure S7A**). The predicted probabilities are in good agreement with the actual 1- to 5-year overall survival rates of glioma patients (**Figures S7B–E**). At the same time, the Kaplan–Meier survival curve was used to demonstrate the good discrimination of survival probability of the two nomogram score groups (**Figure S7F**). Finally, we used the ROC curve to confirm the discriminative ability of this nomogram (AUC = 0.849, **Figure S7G**).

### Validation of DRG2 as a potent therapeutic predictor

**Figure 7:**
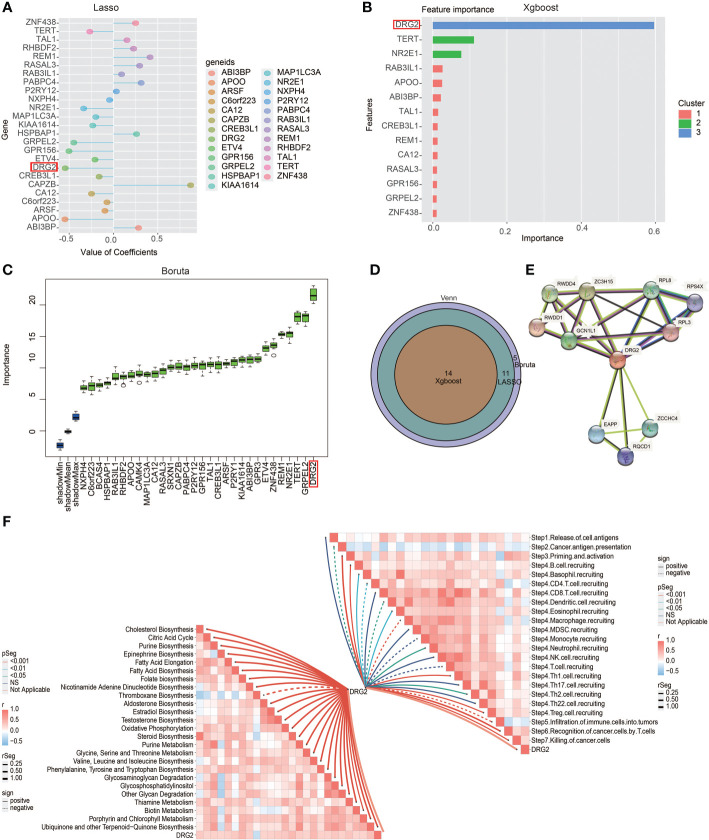
Excavation of characteristic genes using machine learning. **(A)** Characteristic genes between the two clusters defined by LASSO-LR algorithm. **(B)** Characteristic genes between the two clusters defined by Xgboost algorithm. **(C)** Characteristic genes between two clusters defined by Boruta algorithm. **(D)** Venn diagram showing the intersection of three machine learning algorithms. **(E)** Protein–protein interaction analysis showing the interplays of DRG2-related proteins. **(F)** Butterfly plot showing the correlation between DRG2 and metabolism as well as cancer immunity cycle.

**Figure 8:**
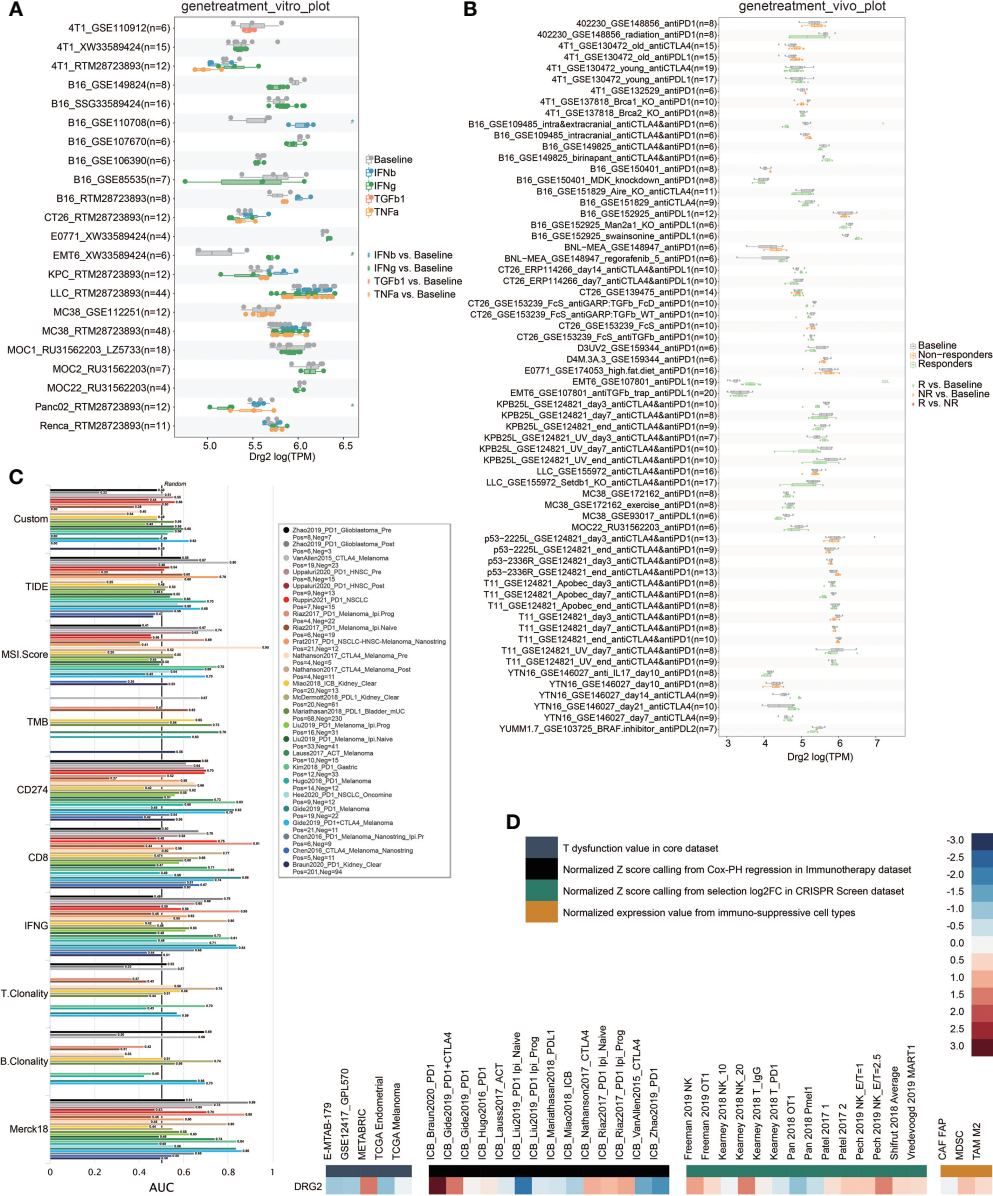
Predictive value of DRG2 in treatment response. **(A)** Predictive value of DRG2 in cytokine treatment. **(B)** Predictive value of DRG2 in immunotherapy cohorts. **(C)** Comparison of DRG2 and conventional immunotherapy predictors in immunotherapy cohorts. **(D)** Heatmap showing the correlation between DRG2 and T dysfunction value in the core dataset, normalized *Z* score calling from Cox-PH regression in the Immunotherapy dataset, normalized *Z* score calling from selection log2FC in the CRISPR Screen dataset, and normalized expression value from immuno-suppressive cell types.

In order to obtain the characteristic gene to well distinguish these two clusters, we then conducted machine learning and prediction. Twenty-nine genes were used as the input for three machine learning algorithms, including LASSO-LR, Xgboost, and Boruta. The feature importance of the powerful genes of the Xgboost algorithm was classified into three clusters. The coefficient values of the powerful genes of the LASSO algorithm were exhibited. The feature importance of the powerful genes of the Boruta algorithm was exhibited. The intersected most powerful prognostic genes identified from the three algorithms were exhibited using the Venn plot. Through LASSO-LR, Xgboost, and Boruta machine learning algorithms, we screened out 25, 14, and 30 genes, respectively (**Figures 7A–C**). Then, we utilized Venn diagram and obtained an intersection of these three algorithms including 14 genes (**Figure 7D**). Among these genes, DRG2 displayed the strongest potency as a characteristic gene, and protein–protein interaction analysis showed the interplays of DRG2-related proteins (**Figure 7E**). Further analysis demonstrated that DRG2 positively correlated with multiple steps in anti-tumor immune response, including recruitment of CD8+ T cell, NK cell, Th1 cell, and Th 17 cell, as well as recognition and killing of cancer cells (**Figure 7F**). DRG2 could predict cytokine treatment response in three cohorts (**Figure 8A**) and immunotherapy response in two cohorts (**Figure 8B**). Furthermore, we compared the immune response of 25 human immunotherapy cohorts between DRG2 and selected conventional biomarkers to better understand the predictive value of DRG2 for immunotherapy. As a result, DRG2 had an AUC > 0.5 in 9 out of 25 cohorts, showing a higher predictive value than TMB and B clonality (**Figure 8C**). In addition, the correlations between DRG2, T-cell dysfunction, and normalized *Z* score are displayed in **Figure 8D**. Furthermore, DRG2 was found to potentially predict the drug response of temozolomide in GBM patients (**Figure S8**).

## Discussion

The role of mast cells remains controversial in TME (16). Mast cells may play different roles in TME, which are related to the type and stage of tumor (14). Some studies found a strong association between mast cells and cancer cell infiltration and tumor angiogenesis as a source of VEGF α, TGF-β, and CXCL8 (29), which means mast cells are related to poor prognosis. On the other hand, mast cells also play an anti-tumor role in tumors (30–32). Some studies found an increase of mast cell in glioma sample and a higher level of mast cell in higher-grade glioma sample (33). Mast cells can be recruited by some factors released by glioma cells, then secrete some macrophage-attracting factors (16, 34). Glioma cells can transform macrophage into glioma-associated macrophages (GAMs), which facilitate tumor proliferation, survival, and migration (35). Therefore, in many human malignant tumors, mast cells are recognized as a key component of TME (36). Notably, mast cell was also proved to affect the prognosis of glioma (37, 38). *Via* consensus clustering, glioma patients were divided into two groups based on mast cell-related genes with unique clinical and immune characteristics from the TCGA, CGGA 325, CGGA 693, and CGGAarray datasets. Compared with patients in cluster 2, patients in cluster 1 had a lower survival probability and a worse prognosis. In different types and grades of gliomas, higher levels of IDH WT, MGMT promoter unmethylation, and 1p19q noncodeletion are more common in high-grade gliomas and the subtype of ME whose prognosis is worse (39). These are associated with a more malignant glioma phenotype with worse prognosis. In addition, patients in cluster 1 were more associated with hypoxia and hypermetabolism, both of which were associated with the malignancy of gliomas.

The classical immune checkpoint molecules such as PD1 and PDCD1LG2 were highly expressed in cluster 1 in the study of tumor immune microenvironment in two clusters of patients. Meanwhile, we found that patients in cluster 1 showed high expression of HLA molecules. In addition, BTN3A1, CXCL9, SLAMF7, TNFRSF4 (OX40), CD27, CD28, and ICOSLG were highly expressed in cluster 1. All of the molecules above are co-stimulators or receptors that increase T-cell proliferation and activation (40–42). As a negative regulator of T-cell activation, the expression of VTCN1 was decreased in cluster 1 (43). This may be the reason for the increased activity of T cells in the glioma patients in cluster 1. This is evidenced by the increased expression of GZMA, which is associated with the pyrotic cell-killing function of the CTL (44).

In previous studies, T-cell activation usually predicted a better prognosis (45). Nevertheless, in the present study, patients in cluster 1 with poor prognosis showed excessive activation of T cells, which may be related to the high-level expression of CCL5 caused by the contact of activated T cells with microglia. Previous studies have shown that CCL5 played an indispensable role in the formation of glioma cells (46). The high-level expression of CCL5 may support immune escape and metastasis of glioma cells (47). CXCL9 can bind to CXCR3 expressed in tumor cells to recruit CD4 + T cells, thus promoting the production of CCL5 in TME, promoting tumor invasion (48). Moreover, the reduction of TNF-α may inhibit the transformation of Th1 to CTL, thereby reducing the ability to kill tumor cells (49). Therefore, we believe that activated T cells in cluster 1 played a more important role in promoting the production and invasion of glioma cells rather than promoting tumor cell apoptosis.

In the investigation of the components of tumor immune infiltrating cells in the two clusters, we found another interesting phenomenon. In previous studies, due to the anti-inflammatory and the promotion of tissue cell repair effects, M2-type macrophages promoted tumor invasion and angiogenesis in the development of gliomas (50). The infiltration of M1-type macrophages that play a pro-inflammatory effect often indicates a better prognosis (51). However, this study showed that glioma patients in cluster 1 had lower M2 infiltration while the infiltration of M1 was higher. Contrary to the results of immune infiltrating cell components, the pro-inflammatory cytokines IL-1β and TNF-α, which was expected to be highly expressed on M1, as well as TLR4, which promoted the differentiation of macrophages to M1 were all lowly expressed in cluster 1 (52, 53). Therefore, we hypothesized that the increased M1 in cluster 1 did not have normal pro-inflammatory effects and tumor-killing functions. The macrophages in cluster 1 may be removed from tumor-killing activity and transformed into GAMs promoting glioma (35). Studies have shown that GAMs could promote tumor growth by secreting immunosuppressive factors and other factors that supported tumor invasion (54). The decreased expression of TNF-α in cluster 1 may represent the impaired function of M1 in GBM, and the increased expression of IL-1β promotes the proliferation and migration of GAMs (39, 55, 56). The gene expression pattern of GAMs is similar to those of all of M0-type, M1-type, and M2-type macrophages (57). Comparing the molecular expression pattern of GAMs in this study with that of GAMs in previous studies, it was more similar to cluster 1 (high levels of IL-12A, CXCL10, VEGFA, and CCL5, and low levels of TLR4) than to cluster 2. CXCL10 promotes the proliferation of GAMS, and the elevated level of VEGFA promotes tumor angiogenesis (58, 59). All these immune molecules were highly expressed in cluster 1. Therefore, the worse prognosis of patients in cluster 1 may be related to the transformation of macrophages into GAMs. However, to prove that macrophages of gliomas in cluster 1 have been transformed into GAMs with tumor-supporting effects, it was necessary to further compare the gene expression pattern of them both. In addition, the low expression of CX3CL1 in cluster 1 may increase tumor invasiveness and promote tumor growth (60). The expression of EDNRB, which has anti-tumor effect, was downregulated in cluster 1 (61). The increased expression of ICAM1 and ITGB2, which mediate cell adhesion, may lead to enhanced tumor aggressiveness (62).

Based on differentially expressed genes (DEGs) between the two clusters, the risk score was calculated based on 29 mast cell-related genes. Mast cell-related risk scores were highly effective in predicting the survival rates of patients at 1, 3, 4, and 5 years. Inclusion of a mast cell-associated risk score with the nomogram further confirmed the effectiveness of mast cells as a prognostic marker.

Next, we tried to establish a relationship between the risk score and TME. Consistent with previous results, all kinds of macrophages increased in the high risk score cluster, suggesting the possibility of the presence of GAMs. The more of Th2 and the less of Th1 in the high risk score cluster indicated that the cellular immunity may be suppressed, and there may be more CCL5 to support the formation of gliomas. As for related immune molecules, consistent with cluster 1, the high expression of T-cell co-stimulation molecules (CD28, ICOSLG, CD27, and CD40) and HLA suggested that the high risk score cluster had a superior activation of T cells and a higher expression of CCL5. The high expression of TGF-β, VEGFA, and CXCL10 and the low expression of TNF-α indicated the tumor-supporting effect of GAMs in high risk score group. Therefore, the tumor immune microenvironment of gliomas with a high risk score overlapped with the gliomas in cluster 1.

In this unprecedented era of big data, there is a wealth of information hidden in huge amounts of data, waiting to be mined and used properly. Machine learning is the scientific discipline focusing on how computers learn from data (63); with its help, models constructed based on clinical information would in return make huge contributions to clinical practice. Our analysis identified a mast cell gene signature consisting of 29 mast cell-specific genes and determined the prognostic value of mast cells in glioma. Our findings proved that mast cells might be a potent factor in stratifying glioma patients’ outcomes. However, the relationship between the polarization of GAMs, the activation of T cells, and the mast cell-related genes in the TME of glioma remains to be further explored. The potential regulatory role of mast cells in the immune response is to be elucidated.

## Data availability statement

The datasets generated and analyzed during the current study are available in the Gene Expression Omnibus (https://www.ncbi.nlm.nih.gov/geo/), TCGA data source (https://xena.ucsc.edu) and CGGA data portal (http://www.cgga.org.cn). The original data has been uploaded to China National Center for Bioinformation (ID: HRA001618). Further inquiries can be directed to the corresponding authors.

## Ethics statement

Glioma tissues were collected and written informed consent was obtained from all patients to participate in the study. The included glioma tissues were approved by the Ethics Committee of Xiangya Hospital, Central South University.

## Author contributions

RZ, HZ, QC, YW, YZ, TL, LZ, and WW designed and drafted the manuscript; HZ, QC, YW, YZ, TL, ZD, XZ, PL, JZ, ZL, and ZW wrote figure legends and revised the article; QC, HZ, and ZD conducted the data analysis. All authors read and approved the final manuscript. All authors contributed to the article and approved the submitted version.

## Funding

This work was supported by the National Natural Science Foundation of China (82073893), Hunan Provincial Natural Science Foundation of China (2022JJ20095, 2019JJ80056), Hunan Provincial Health Committee Foundation of China (202204044869).

## Conflict of interest

The authors declare that the research was conducted in the absence of any commercial or financial relationships that could be construed as a potential conflict of interest.

## Publisher’s note

All claims expressed in this article are solely those of the authors and do not necessarily represent those of their affiliated organizations, or those of the publisher, the editors and the reviewers. Any product that may be evaluated in this article, or claim that may be made by its manufacturer, is not guaranteed or endorsed by the publisher.

## Abbreviations

CDF, cumulative distribution function; CGGA, Chinese Glioma Genome Atlas; CL, classic; CNA, copy number alternations; CNV, copy number variation; CR, complete response; DEG, differentially expressed gene; GAM, glioma-associated microglia/macrophages; GBM, glioblastoma; GO, gene ontology; GSVA, gene set variation analysis; LGG, low-grade glioma; ME, mesenchymal; NE, neural; PAM, partition around medoids; PCA, principal component analysis; PD, progressive disease; PN, pro-neural; PR, partial response; SD, stable disease; TAM, tumor-associated macrophage; TCGA, The Cancer Genome Atlas; TME, tumor microenvironment.
