# Supplementary material for: Large-scale bulk RNA-seq analysis defines immune evasion mechanism related to mast cell in gliomas
Source: Front Immunol. 2022 Sep 8;13:914001. doi: 10.3389/fimmu.2022.914001 (PMC9492887; doi:10.3389/fimmu.2022.914001)
Supplement: Supplementary file 1 [file DataSheet_1.docx]

**Figure legends**

**
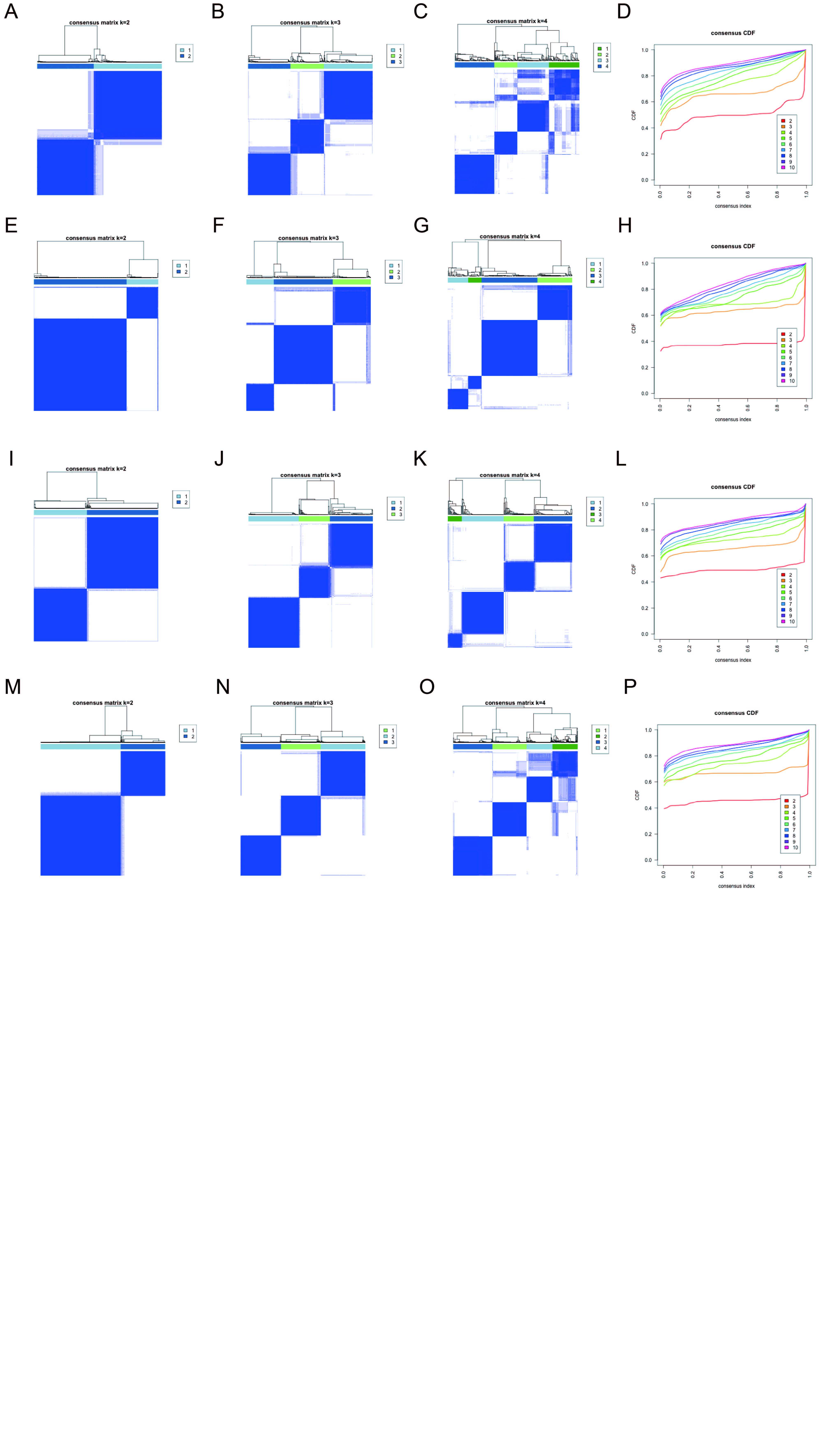
**

**Figure S1.** Clustering stability analysis by ConsensusClusterPlus package. Using data derived from A-D. CGGA325, E-H. CGGA693, I-L. CGGAarray and M-P. TCGA.


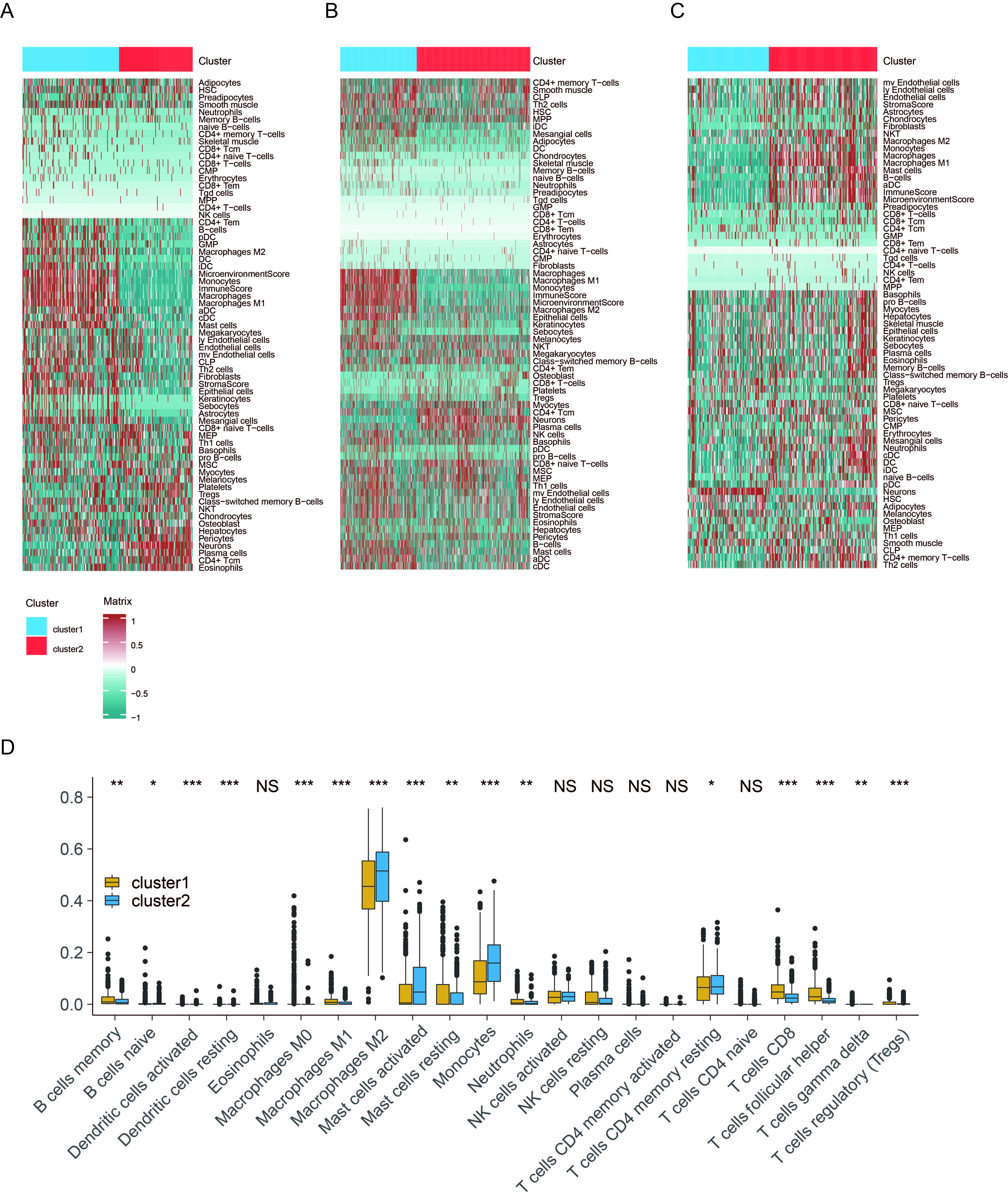


**Figure S2.** Dendrogram correlating the levels of 64 cell types and clusters in A. CGGA325, B. CGGA693 and C. CGGAarray. D. Expression level of 22 immune cell types in two clusters in TCGA.


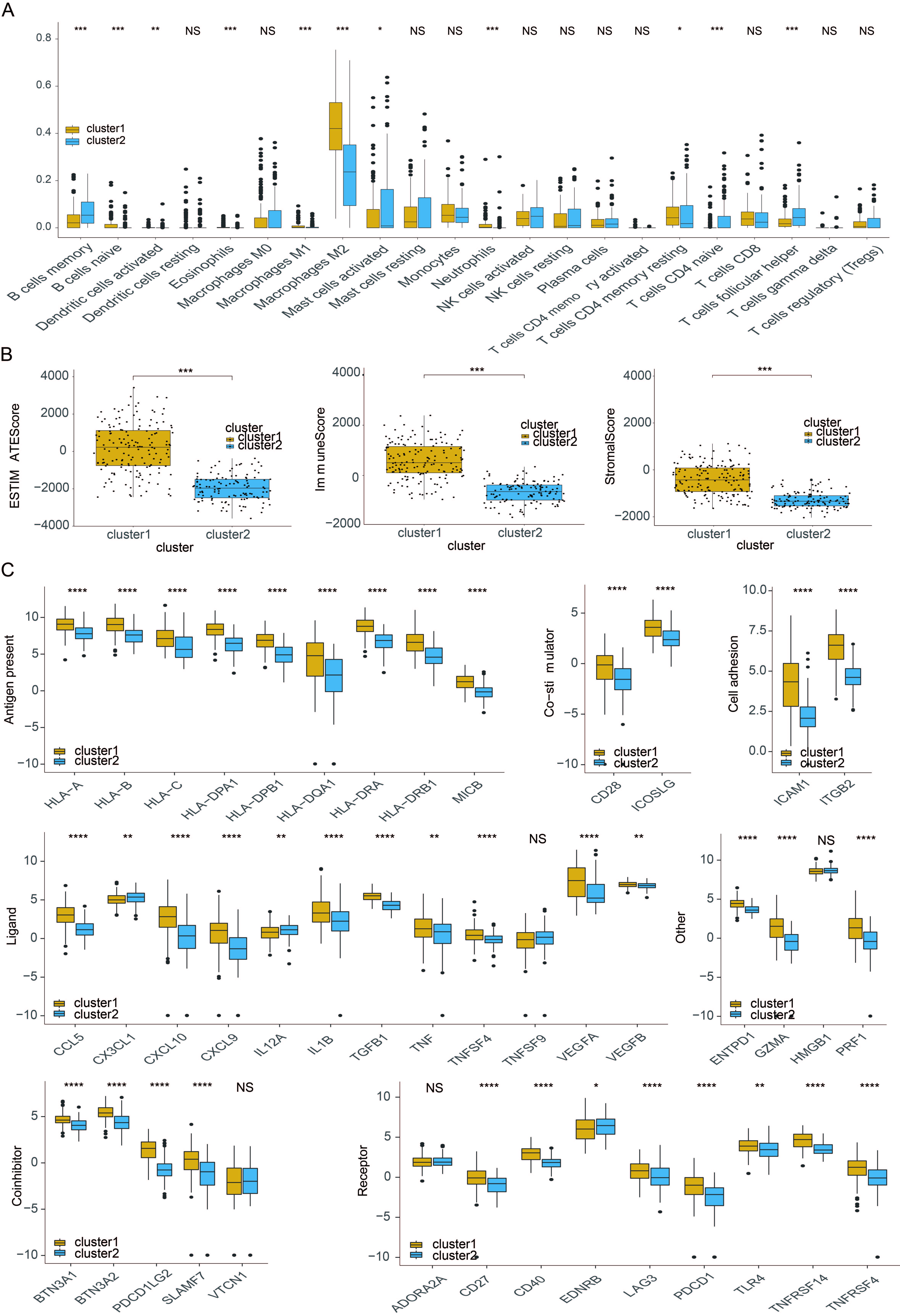


**Figure S3.** A. Expression level of 22 immune cell types in two clusters in CGGA325. B. ESTIMATEScores, ImmuneScores and StromalScores of the two clusters in CGGA325. C. Molecule levels of immune checkpoints in two clusters in CGGA325.


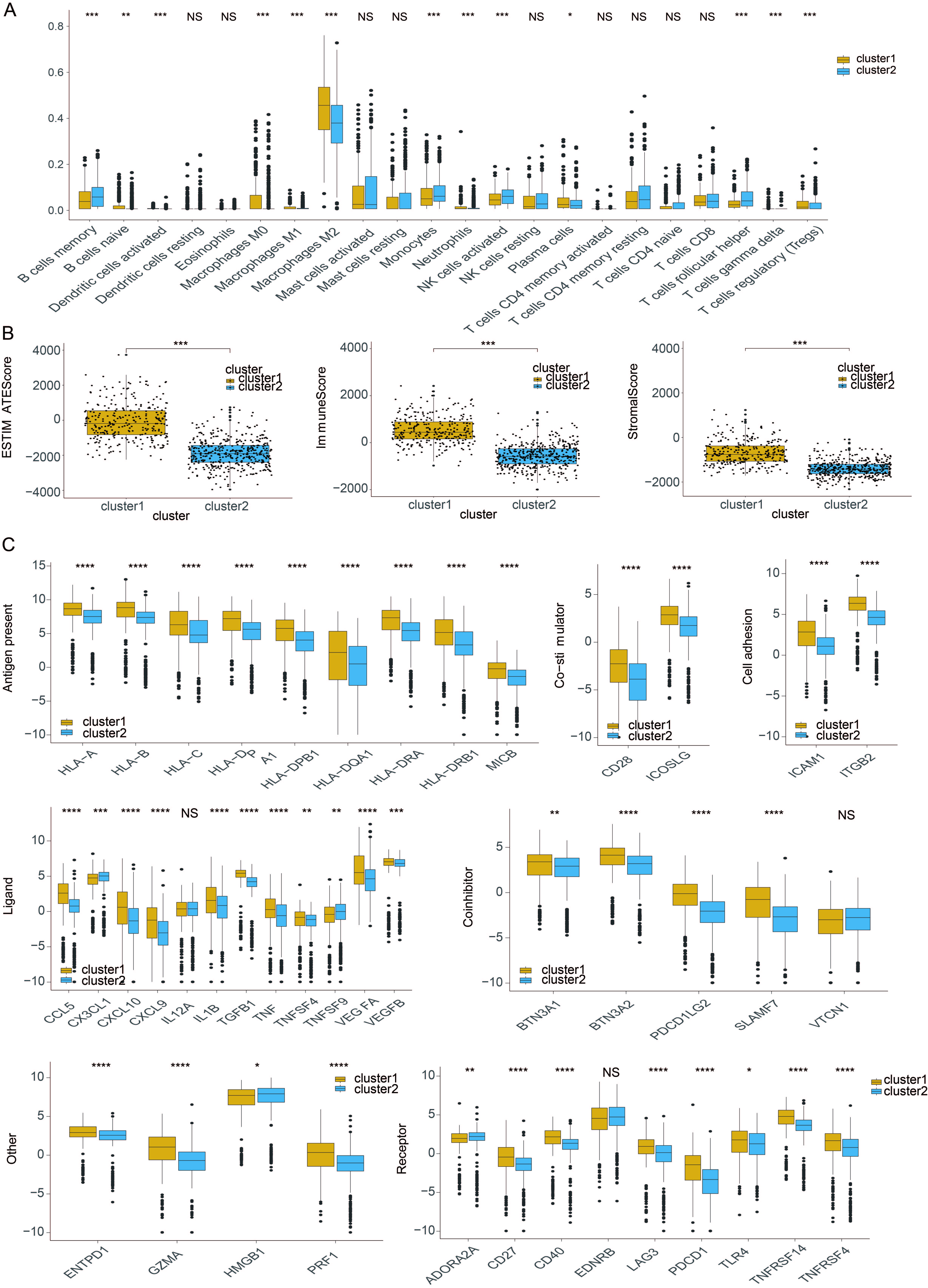


**Figure S4.** A. Expression level of 22 immune cell types in two clusters in CGGA693. B. ESTIMATEScores, ImmuneScores and StromalScores of the two clusters in CGGA693. C. Molecule levels of immune checkpoints in two clusters in CGGA693.


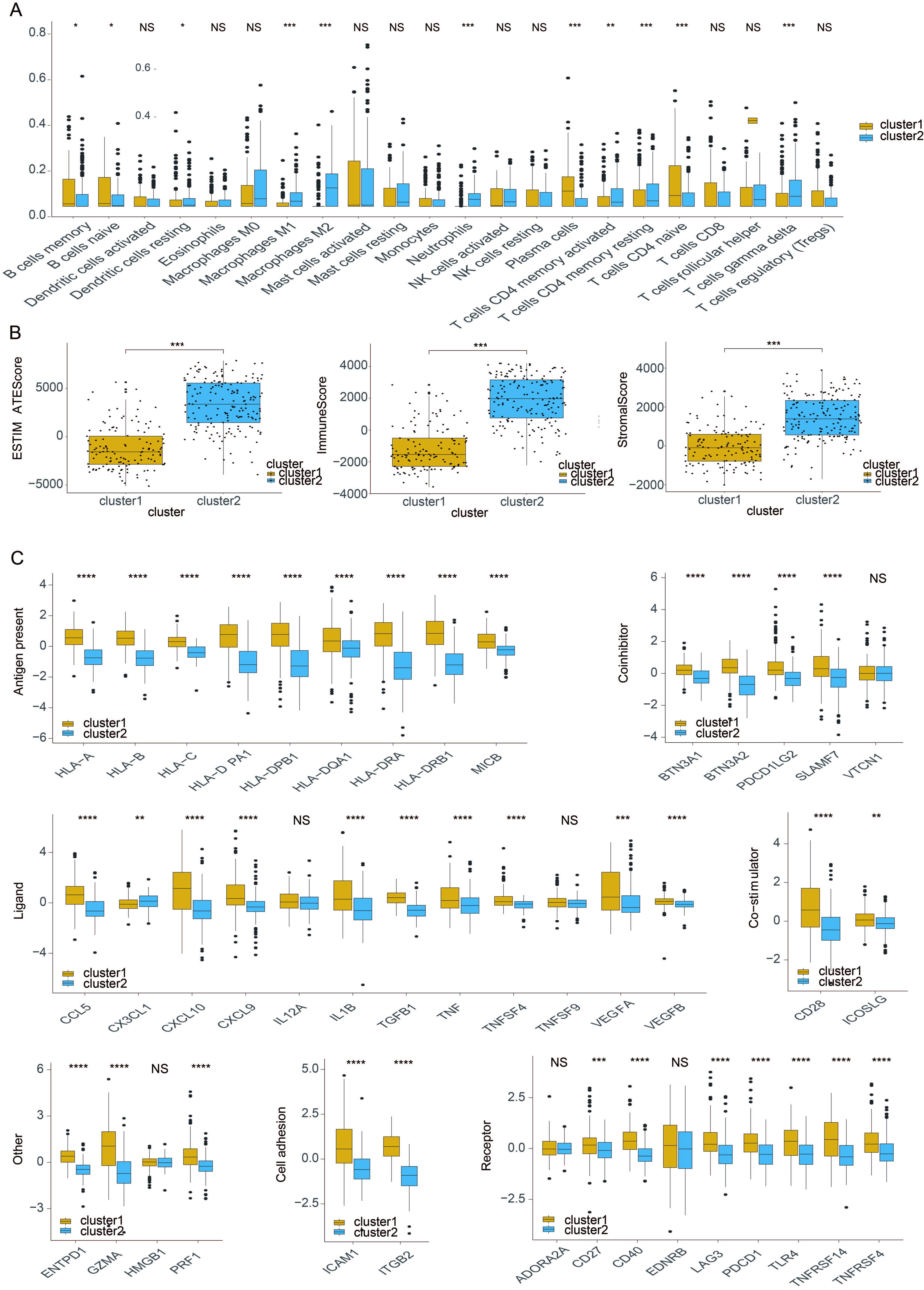


**Figure S5.** A. Expression level of 22 immune cell types in two clusters in CGGAarray. B. ESTIMATEScores, ImmuneScores and StromalScores of the two clusters in CGGAarray. C. Molecule levels of immune checkpoints in two clusters in CGGAarray.


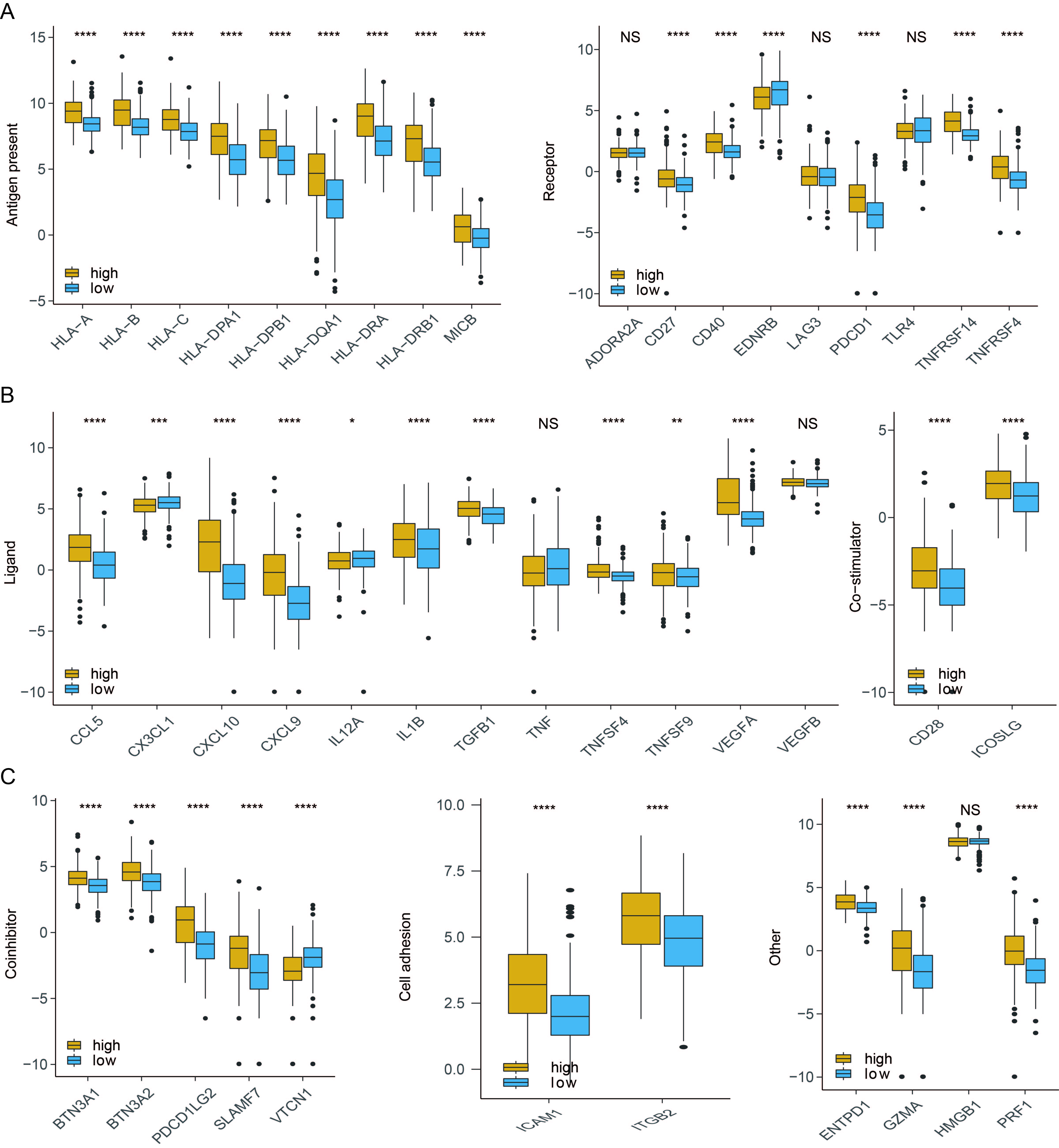


**Figure S6.** Molecule levels of immune checkpoints in high and low risk scores in TCGA.


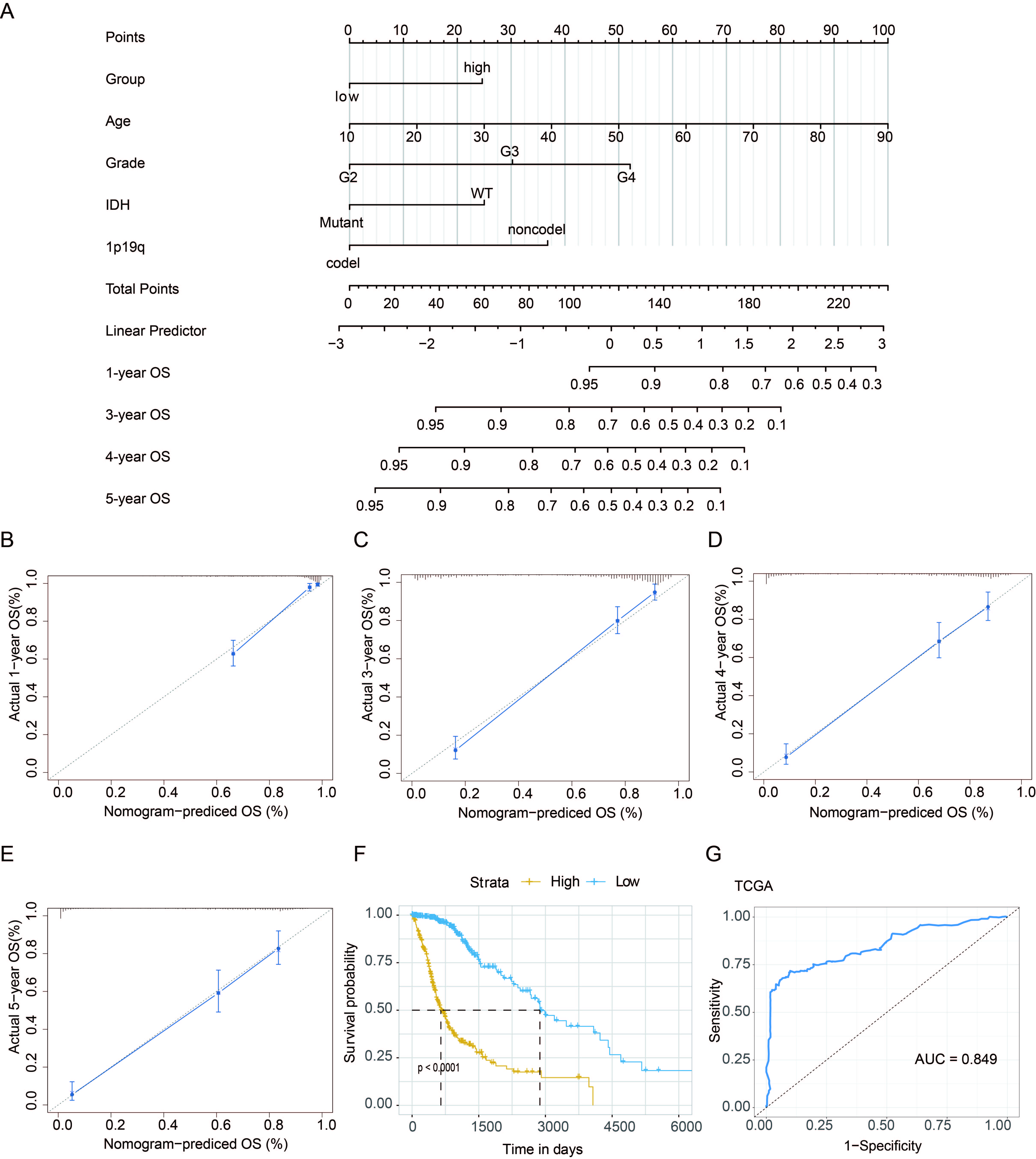


**Figure S7.** Clinical model of the two clusters. A. Nomogram for predicting the proportion of glioma patients with OS. Comparison between the nomogram-predicted OS and actual B. 1-year, C. 3-year, D. 4-year, E.5-year OS in TCGA. F. Kaplan-Meier analysis of OS based on high vs low level of mast cell in TCGA. G. The ROC curve to assess sensitivity and specificity of two clusters as a diagnostic biomarker in gliomas.


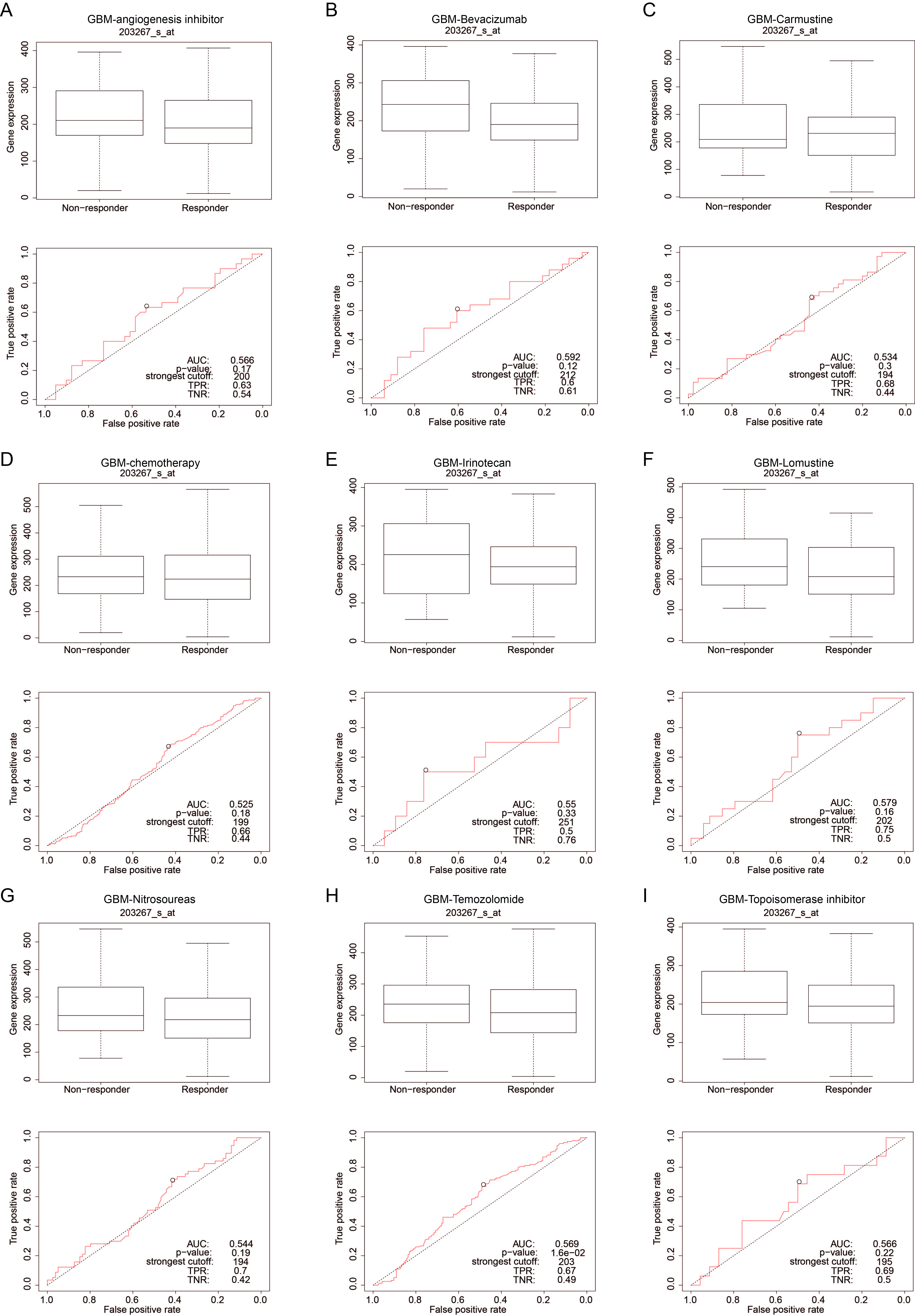


**Figure S8.** The predictive value of DRG2 in drug response. Box plot showing the expression value of DRG2 in non-responders and responders in GBM patients receiving A. angiogenesis inhibitor, B. Bevacizumab, C. Carmustine, D. chemotherapy, E. Irinotecan, F. Lomustine, G. Nitrosoureas, H. Temozolomide, I. Topoisomerase inhibitor. ROC curve showing the predictive value of DRG2 in nine treatment modalities, respectively.
